# *Mycobacterium intracellulare* Infection Mimicking Progression of Scleroderma

**DOI:** 10.1155/2017/4029271

**Published:** 2017-09-27

**Authors:** Simon Krabbe, Merete Engelhart, Sören Thybo, Søren Jacobsen

**Affiliations:** ^1^Center for Rheumatology and Spine Diseases, Rigshospitalet, 2600 Glostrup, Denmark; ^2^Center for Rheumatology and Spine Diseases, Gentofte Hospital, 2900 Hellerup, Denmark; ^3^Department of Infectious Diseases, Rigshospitalet, 2100 Copenhagen, Denmark; ^4^Center for Rheumatology and Spine Diseases, Rigshospitalet, 2100 Copenhagen, Denmark

## Abstract

This case report describes a patient with scleroderma who developed *Mycobacterium intracellulare* infection, which for more than a year mimicked worsening of her connective tissue disorder. The patient was diagnosed with scleroderma based on puffy fingers that developed into sclerodactyly, abnormal nail fold capillaries, interstitial lung disease, Raynaud's phenomenon, esophageal dysmotility, and positivity for rheumatoid factor and anti-SSA antibodies. She developed massive inflammatory changes of the cutis, the subcutis, and the muscle fasciae of the right leg, that after several failed attempts of immunosuppressive treatments were found to be caused by *Mycobacterium intracellulare*. While she was receiving high-dose prednisolone, as worsening of her connective tissue disease was suspected to be the cause of the inflammatory changes, she had *Listeria monocytogenes* meningitis and was hospitalized for several weeks, but she recovered from this without sequelae. After *Mycobacterium intracellulare* infection was diagnosed, she was treated with clarithromycin and rifampicin. Her skin manifestations, arthralgias, and fatigue improved considerably, and the wounds of the right leg healed, unfortunately with significant scarring. Immunodeficiency testing was unremarkable. In summary, an infection with *Mycobacterium intracellulare* was mistaken for an unusually severe progression of scleroderma.

## 1. Introduction

We describe a patient with scleroderma who developed patchy, indurated elements of her skin, fasciitis, and arthritis that was long thought to represent activity of her rheumatic disease that had long been hard to classify. The manifestations progressed despite therapy with high-dose prednisolone. Skin biopsy showed granulomatous inflammation caused by *Mycobacterium intracellulare*. Remarkably, she had progression of scleroderma in parallel with mycobacterial infection that mimicked cutaneous manifestations of connective tissue disease.

## 2. Case Presentation

This patient developed thickened and painful fingers, dyspnea, arthritis of the knees, and a painful swelling dorsal to the right knee with redness of the skin at age 41 years. She had a history of myalgias with elevated C-reactive protein but with normal creatine kinase and a painful necrotic wound at the medial malleolus of her left ankle in the preceding years; these problems had responded well to prednisolone and leflunomide as steroid-sparing agent. Deep venous thrombosis was excluded with ultrasonography. A high-resolution CT scan of the lungs showed subpleural reticulation mainly of the lower lobes, discrete ground glass changes, and traction bronchiectasis. She had low forced expiratory volume after one second, 2.5 L (77% of expected), and low total lung capacity, 4.7 L (82% of expected), but normal alveolar volume-corrected carbon monoxide diffusion, 1.5 mmol/min/kPa/L (93% of expected). Bronchoalveolar lavage and transbronchial biopsies showed no fungal infection. Hep-2 cell anti-nuclear antibody testing showed a nuclear speckled pattern, and anti-SSA antibodies were present in high titer. She had eosinophilia, 0.52 × 10^9^/L, and elevated immunoglobulin G (IgG), 33 g/L.

The skin elements of the right leg progressed, as did the tightness of the skin of her fingers. On low suspicion of erysipelas or Lyme disease, she was treated with benzylpenicillin and doxycycline without effect. Scleroderma, eosinophilic fasciitis, and systemic lupus erythematosus were considered. A skin biopsy of the right leg showed deep subcutaneous and fascial inflammation, both perivascular and panniculitis, with nodular lymphocytic infiltrates, many plasma cells, a few eosinophils, and a few granulomas. It was judged compatible with, but not specific for, eosinophilic fasciitis. A bone marrow biopsy showed hyperplastic myelopoiesis similar to what had been observed years earlier. Radiography of the esophagus showed dysmotility of the lower third compatible with scleroderma. The arthralgias in knees and shoulders worsened, and she developed livedo reticularis, malaise, and loss of appetite.

Methotrexate was prescribed, but the skin elements worsened, and she was admitted with abdominal pain. Enlarged mediastinal and axillary lymph nodes were found by computed tomography. Inflammatory bowel disease was searched for but dismissed after gastroscopy, colonoscopy, and magnetic resonance imaging (MRI) of duodenum and jejunum. Severe *Candida* esophagitis was diagnosed and treated. Then, while on prednisolone 22.5 mg qd, she was hospitalized because of *Listeria monocytogenes* meningitis for three weeks, but she recovered from this without sequelae.

**Figure 1:**
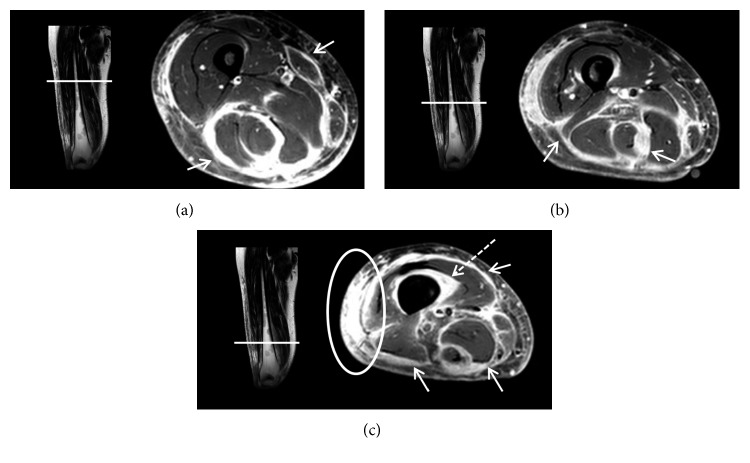
Coronal STIR and axial T1-weighted postcontrast SPIR magnetic resonance images of the upper (a), middle (b), and lower (c) right thigh. The images show considerable and diffuse inflammatory changes along the fasciae of all muscles of the right lower thigh (arrows) and surrounding the cortical bone of the distal femur (dotted arrow), and severe inflammatory changes surrounding the vessels of the popliteal fossa and subcutaneously at the knee level extending cranially along the lateral side of the thigh (circle).

**Figure 2:**
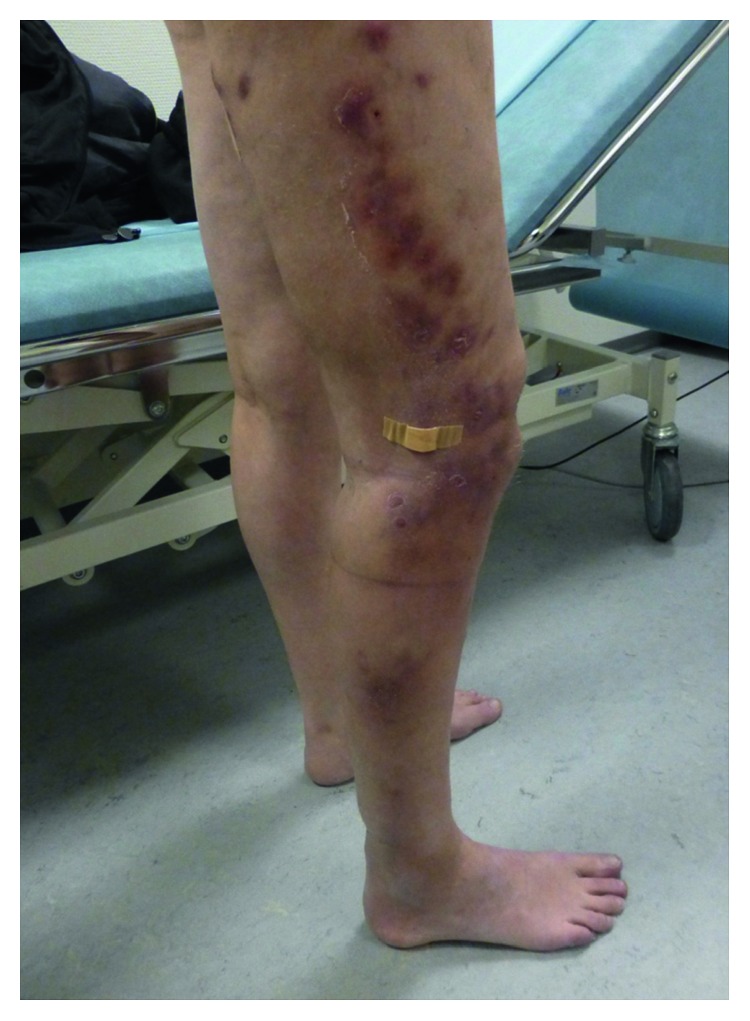
Photography of the patient with bluish red indurated patches of the right lower leg extending up through the lateral part of the thigh, with swelling of the entire right leg. The patient had covered an ulcerating element with band aid.

At age 43 years, MRI (Figure 1()) showed considerable inflammatory changes along the fasciae of all muscles of the distal right thigh and severe subcutaneous contrast enhancement. At this stage, over a year after the first hard skin elements of the right lower leg had emerged, the skin changes had progressed from the lower leg up through the thigh, and the clinical presentation of the patient was suggestive of an infectious etiology (Figure 2()).

**Figure 3:**
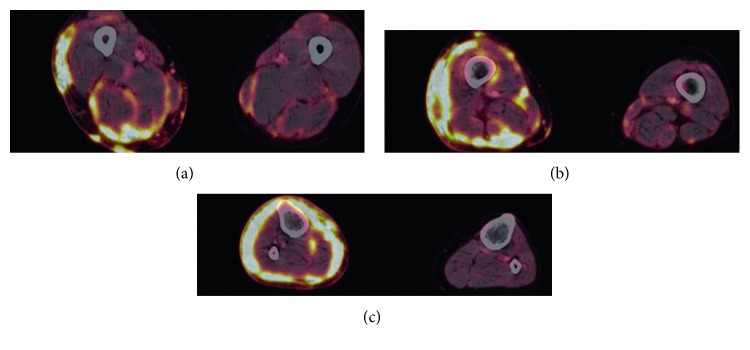
PET-CT showed pronounced inflammatory cutaneous changes of the entire circumference of the right leg and of fasciae surrounding several muscles. Minor inflammatory changes of the subcutis and muscle fasciae of the left leg are also present. (a) Upper part of thigh. (b) Lower thigh. (c) Upper part of lower leg.

A repeated skin biopsy revealed severe granulomatous inflammation of epithelioid cells with small necroses. The Ziehl-Neelsen stain showed acid-fast bacilli, and culturing identified them as *Mycobacterium intracellulare*, one of the two species of *Mycobacterium avium* complex (MAC) within the group of nontuberculous mycobacteria. A PET-CT scan (Figure 3()) showed pronounced inflammatory cutaneous changes of almost the whole right leg with possible muscular involvement, patchy inflammatory changes of the muscles and skin of the left leg, lymphadenitis of the inguinal and iliac lymph nodes, and minor skin changes of the right upper arm similar to those of the right leg.

She responded well to 12-month treatment with clarithromycin and rifampicin, the swelling of the leg diminished gradually, her arthralgias and malaise improved, and the wounds of her right leg healed slowly, unfortunately with significant scarring. One year after finishing this treatment, she was feeling well and had no significant dyspnea or difficulty swallowing, but the skin changes of her fingers compatible with scleroderma persisted and SSA antibodies remained high-titer positive, while anti-RNA-polymerase III, anti-centromere, and anti-SCL-70 antibodies were negative.

## 3. Discussion

Scleroderma was judged as a reasonable diagnosis based on her puffy fingers that developed into sclerodactyly, abnormal nail fold capillaries, Raynaud's phenomenon, interstitial lung disease, and lower esophageal dysmotility [1]. We wondered why she contracted two rather unusual pathogens, *Listeria* and *Mycobacterium*, that share the propensity to live inside the host's cells and are guarded against by the cellular immune response [2, 3]. Immune-deficiency testing showed normal levels of immunoglobulins, normal vaccination response to diphtheria and tetanus, low levels of CD8-positive T cells, normal levels of CD4-positive T cells and NK cells, and normal IL-12 and interferon-γ signalling [4]. The immunosuppressive treatments and the presence of an autoimmune disease were possible causal factors. This case report demonstrates that *Mycobacterium intracellulare* infection is a possible differential diagnosis when a patient with established scleroderma has cutaneous manifestations that are unusually severe and progressing despite immunosuppressive therapy.

## References

[1] van den Hoogen F., Khanna D., Fransen J. (2013). 2013 classification criteria for systemic sclerosis: an American College of Rheumatology/European League Against Rheumatism collaborative initiative. *Annals of the Rheumatic Diseases*.

[2] Henkle E., Winthrop K. L. (2015). Nontuberculous mycobacteria infections in immunosuppressed hosts. *Clinics in Chest Medicine*.

[3] Calame D. G., Mueller-Ortiz S. L., Wetsel R. A. (2016). Innate and adaptive immunologic functions of complement in the host response to *Listeria monocytogenes* infection. *Immunobiology*.

[4] Weiss G., Schaible U. E. (2015). Macrophage defense mechanisms against intracellular bacteria. *Immunological Reviews*.

